# *Hippeastrum reticulatum* (Amaryllidaceae): Alkaloid Profiling, Biological Activities and Molecular Docking

**DOI:** 10.3390/molecules22122191

**Published:** 2017-12-09

**Authors:** Luciana R. Tallini, Edison H. Osorio, Vanessa Dias dos Santos, Warley de Souza Borges, Marcel Kaiser, Francesc Viladomat, José Angelo S. Zuanazzi, Jaume Bastida

**Affiliations:** 1 Group of Natural Products, Faculty of Pharmacy, University of Barcelona, Av. Joan XXIII, 27-31, 08028-Barcelona, Spain; lucianatallini@gmail.com (L.R.T.); fviladomat@ub.edu (F.V.); 2 Department of Basic Sciences, Catholic University Luis Amigó, SISCO, Transversal 51 A No. 67B-90, Medellín, Colombia; edison.osorio@gmail.com; 3 Department of Chemistry, Federal University of Espírito Santo, Av. Fernando Ferrari 514, 29075-915 Vitória ES, Brazil; vanessadds@yahoo.com.br (V.D.d.S.); warley.borges@ufes.br (W.d.S.B.); 4 Medicinal Parasitology and Infection Biology, Swiss Tropical Institute, Socinstrasse 57, 4051 Basel, Switzerland; marcel.kaiser@unibas.ch; 5 University of Basel, Petersplatz 1, 4001 Basel, Switzerland; 6 Faculty of Pharmacy, Federal University of Rio Grande do Sul, Av. Ipiranga 2752, 90610-000 Porto Alegre RS, Brazil; zuanazzi@ufrgs.br

**Keywords:** 6α-hydroxymaritidine, 6β-hydroxymaritidine, reticulinine, isoreticulinine, *Hippeastrum reticulatum*

## Abstract

The Amaryllidaceae family has proven to be a rich source of active compounds, which are characterized by unique skeleton arrangements and a broad spectrum of biological activities. The aim of this work was to perform the first detailed study of the alkaloid constituents of *Hippeastrum reticulatum* (Amaryllidaceae) and to determine the anti-parasitological and cholinesterase (AChE and BuChE) inhibitory activities of the epimers (6α-hydroxymaritidine and 6β-hydroxymaritidine). Twelve alkaloids were identified in *H. reticulatum*: eight known alkaloids by GC-MS and four unknown (6α-hydroxymaritidine, 6β-hydroxymaritidine, reticulinine and isoreticulinine) by NMR. The epimer mixture (6α-hydroxymaritidine and 6β-hydroxymaritidine) showed low activity against all protozoan parasites tested and weak AChE-inhibitory activity. Finally, a molecular docking analysis of AChE and BuChE proteins showed that isoreticulinine may be classified as a potential inhibitory molecule since it can be stabilized in the active site through hydrogen bonds, π-π stacking and hydrophobic interactions.

## 1. Introduction

Natural products offer a wealth of bio-structural information that can be used to guide drug discovery and molecular design projects [1]. Plants have played a huge role in traditional medicine, and plant screening has become an important tool in drug discovery for pharmaceutical companies and university institutes [2]. Alkaloids are plant secondary metabolites that are usually low in nitrogen but high in oxygen [3]. Their three-dimensional complexity and interesting ring systems are of great interest for drug development [3,4,5].

The Amaryllidaceae, a plant family in the monocot order Asparagales, is composed of three subfamilies: Amaryllidoideae, Agapanthoideae and Allioideae [6,7]. The Amaryllidoideae include about 80 genera [8] that are mainly distributed in tropical and subtropical regions, but also in temperate zones [9]. A particular feature of plants of the Amaryllidoideae subfamily is their content of an exclusive, numerous and still expanding group of alkaloids, known as Amaryllidaceae alkaloids. These compounds are characterized by unique skeleton arrangements and a broad spectrum of biological activities [10]. Galanthamine is the most important Amaryllidaceae alkaloid, having received FDA approval in 2001 for the clinical management of mild to moderate Alzheimer’s disease (AD) [11].

Over 47 million people worldwide were living with dementia in 2016, a figure expected to increase to 131 million by 2050 [12]. The most common symptom of AD is a deterioration of memory and other cognitive domains, leading to death within 3 to 9 years after diagnosis [13]. Multiple factors have been implicated in AD pathology, including a reduced cholinergic neurotransmission level, oxidative stress and aggregated amyloid-β-peptide (Aβ) [14].

Another global health challenge is tackling the neglected tropical diseases (NTDs), a diverse group of communicable diseases that prevail in tropical and subtropical conditions and affect more than a billion people [15]. The World Health Organization (WHO) lists 17 NTDs, including Chagas disease, human African trypanosomiasis (sleeping sickness) and leishmaniosis. Alongside malaria, these diseases are highly feared in affected populations, where poverty limits access to prevention and treatment interventions [15]. One of the main reasons why these diseases are “neglected” is that there is little incentive for the pharmaceutical industry to invest in developing drugs for a patient population that cannot afford them [16]. Consequently, much of the research on effective treatment of these diseases is carried out in academic laboratories with limited financial, personnel, and technical resources [16].

The *Hippeastrum* Herb. is a genus (Amaryllidaceae) native to South America and comprises approximately 60 species [17], about 30 of which are found in Brazil; the majority are endemic and poorly studied [18]. This genus has been traditionally used to cure piles, tumors and various inflammatory disorders such as asthma [19]. Physiological activities reported for plants of this genus include psychopharmacological [20], against *Trichomonas vaginalis* [21] and cytotoxicity [22,23]. The species *H. reticulatum* is widely used as an ornamental plant due to its colorful flowers ranging from light pink to dark purple [24]. In recent years, some studies have been published about its primary metabolites, pigment constituents (anthocyanins), botanical characterization, karyotypes and phylogenesis [9,24,25,26].

**Figure 1:**
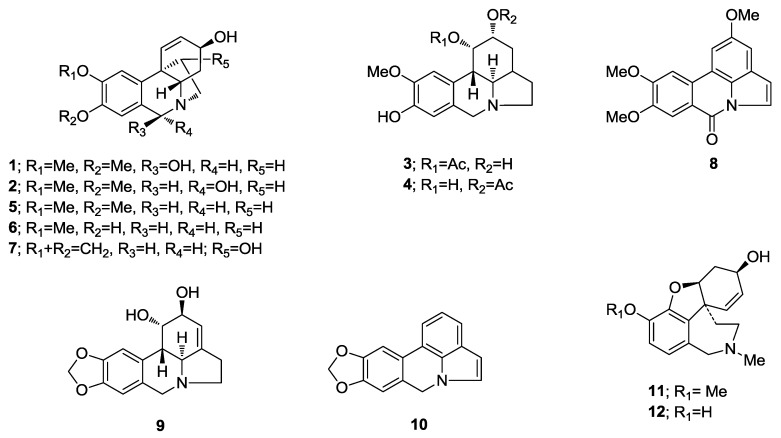
Alkaloids identified in *H. reticulatum*.

The aim of this work was to perform the first study of the alkaloid profile and biological activities of *Hippeastrum reticulatum*. Four new alkaloids were isolated and chemically characterized by spectroscopic methods and eight known alkaloids were identified by gas chromatography-mass spectrometry (GC-MS). Anti-parasitological and cholinesterase—acetylcholinesterase (AChE) and butyrylcholinesterase (BuChE)—inhibitory activities of the epimers (compounds 1 and 2, Figure 1) are described. Molecular docking studies were also carried out to investigate the affinity of the four new compounds for the active sites of AChE and BuChE based on intermolecular features such as hydrogen bonds, π-π stacking and hydrophobic interactions. Unfortunately, it was not possible to determine the biological activities of the isomers (compounds 3 and 4, Figure 1) due to a lack of samples.

## 2. Results and Discussion

### 2.1. Alkaloid Identification by GC-MS

The MeOH extracts of *H. reticulatum* plants were fractioned following the sequence described in the Experimental section. The fractions were analyzed and the structural types of the alkaloids from this species were identified by comparing their GC-MS spectra and Kovats Retention Index (RI) values with those of authentic Amaryllidaceae alkaloids previously isolated and identified by spectrometric methods (NMR, UV, CD, IR, MS) in our laboratory, by the NIST 05 Database or by literature data (Table 1). Eight known alkaloids were identified by GC-MS using our Amaryllidaceae alkaloid library (Figure 1) and four new alkaloids (**1**–**4**) were isolated and chemically characterized by spectroscopic methods.

### 2.2. Structural Elucidation by NMR

#### 2.2.1. 6β-Hydroxymaritidine (**1**) and 6α-hydroxymaritidine (**2**)

**Figure 2:**
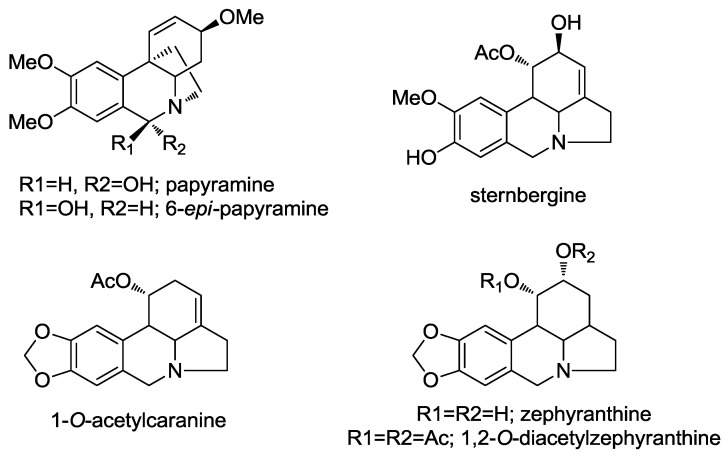
Alkaloids allowing the structural elucidation of compounds **1**–**4**.

**Figure 3:**
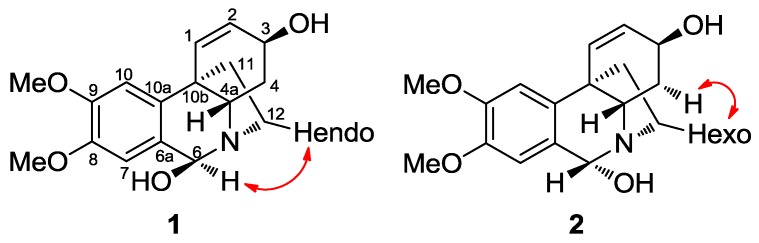
Key NOESY correlations of compounds **1** and **2**.

Compounds **1** and **2** were obtained together from an acetate leaf fraction and isolated as a mixture, which was not separated by conventional chromatographic methods. The HRMS data of compounds **1** and **2** suggested the molecular formula C_17_H_22_NO_4_ for the parent ion [M + H]^+^ at *m/z* 304.1544 (calcd 304.1543). The absolute configuration of these epimers was determined by circular dichroism (CD). The curve and shape were qualitatively similar to those of known haemanthamine-type alkaloids, with the 5,10b-ethano bridge in an α-orientation, having a minimum of 250 nm and a maximum of around 285 nm. The ^1^H-NMR data of compounds **1** and **2** (Table 2) were similar to the data published for papyramine and 6-*epi*-papyramine [27] (Figure 2). The spectrum showed two singlets at *δ* 5.35 and 6.12 due to the benzylic H-6 proton for epimers **1** and **2**, respectively. Two double doublets of the olefinic protons at *δ* 5.98 and 6.02 were assignable to the H-2 protons of both epimers (compounds **1** and **2**, respectively) due to their coupling with the H-1 doublets at 6.59 and 6.51 ppm, respectively. The value of the H-2/H-3 coupling constant (*J* = 5.3 and 5.1 Hz, respectively) was in agreement with a β-orientation of the hydroxyl group at C-3. The assignment of the aromatic protons was made on the basis of the relative intensities of the signals of both epimers and the benzylic coupling between H-6 and H-7, observed in the 2D COSY experiment. Two signals for the methoxy groups at *δ* 3.90 and 3.87 were observed, both of them integrating 4.3 protons (since epimers **1** and **2** were in a ratio of 3:1). The protons H-4a and H-4ax showed a large coupling (*J* = 13.6 and 13.8 Hz, compound **1** and **2**, respectively), due to their trans diaxial configuration. The NOESY contour between H-6α and H-12endo allowed us to assign the β-orientation of the hydroxyl group in compound **1** (Figure 3). The coupling between H-12exo and H-4α allowed us to establish the conformational orientation of these protons in compound **2** (Figure 3). All the signals were confirmed by 2D-NMR.

#### 2.2.2. Reticulinine (**3**) and Isoreticulinine (**4**)

**Figure 4:**
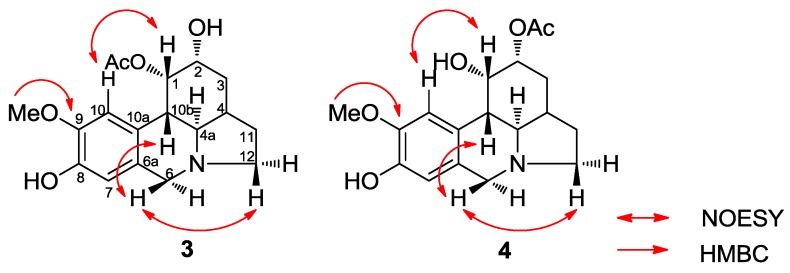
Key NOESY correlations of compounds **3** and **4**.

Compounds **3** and **4** were obtained from an acetate bulb fraction and isolated as a mixture due to limited sample availability. The HRMS data of compounds **3** and **4** suggested the molecular formula C_17_H_22_NO_4_ for the parent ion [M + H]^+^ at *m/z* 334.1651 (calcd 334.1649). The ^1^H-NMR data summarized in Table 3 were similar to those reported for zephyranthine, 1,2-*O*-diacetylzephyranthine [28] and sternbergine [29] and 1*-O*-acetylcaranine [30] (Figure 2). A correlation between the protons of the methoxy group and H-10 in the 2D NOESY spectrum allowed us to assign this group at C-9 in both compounds (Figure 4). The two double doublets at *δ* 3.11 (*J* = 5.9 and 10.4 Hz) and 3.22 (*J* = 5.9 and 10.7 Hz) were attributed to the H-4a position in compounds **3** and **4**, respectively. The large coupling constant supported a *trans*-fusion of the B/C rings in both structures. The location and axial configuration of the radical group at C-1 in compounds **3** and **4**, respectively, were deduced from COSY and NOESY experiments (Figure 4). In compound **3,** the low magnetic field location of the H-1 and H-2 protons (*δ* 6.01 and 4.15 ppm, respectively) in the ^1^H spectrum allowed us to determine the presence of an acetyl group at C-1 and a hydroxyl group at C-2, respectively. Conversely, in compound **4**, the low magnetic field of the H-1 and H-2 protons (*δ* 4.77 and 5.12 ppm, respectively) indicated a hydroxyl group at C-1 and an acetyl group at C-2, respectively. In these compounds, the equatorial H-1 resonated as a triplet at *δ* 6.01 (*J* = 3.9 Hz) and 4.77 (*J* = 1.5 Hz), respectively, and was coupled to H-10b and H-2 in the COSY spectrum, and to H-10 in the NOESY experiment (Figure 4). These data and the large constant coupling between H-2 and H-3α in compounds **3** and **4** (*J* = 9.8 and 11.6 Hz, respectively) allowed us to assign the equatorial configuration of the substituent at C-2. From the HMBC spectrum, three bond correlations were observed for H-7 to C-9, H-10 to C-8, H-7 to C-10a and H-10 to C-6a, enabling us to identify the resonances of the quaternary carbons C-8, C-9, C-6a and C-10a. In addition, a three-bond coupling between the methoxy protons and C-9 confirmed the NOESY result (Figure 4).

### 2.3. Biological Activity

#### 2.3.1. Antiprotozoal Activity

6β-Hydroxymaritidine (**1**) and 6α-hydroxymaritidine (**2**) were isolated as an epimer mixture, which showed low activity against all protozoan parasites tested (Table 4). However, this sample also presented low cytotoxicity (>100 µg mL^−1^), so it could be interesting to isolate the epimers and analyze their individual antiprotozoal activity. The quantity of reticulinine (**3**) and isoreticulinine (**4**) was insufficient for an antiprotozoal study.

#### 2.3.2. Acetylcholinesterase and Butyrylcholinesterase Inhibitory Activities

6β-Hydroxymaritidine (**1**) and 6α-hydroxymaritidine (**2**), isolated as an epimer mixture, were tested for in vitro AChE- and BuChE-inhibitory activities. With galanthamine as a positive control, the epimers exhibited weak AChE inhibition (IC_50_ 90.43 ± 4.26 µM) and no BuChE inhibition (IC_50_ > 600 µM), while galanthamine presented AChE and BuChE inhibition (IC_50_ 1.56 ± 0.14 and 12.96 ± 0.65 µM, respectively). As described below in the molecular docking results, it would be of interest to check the cholinesterase inhibitory activities of reticulinine (**3**) and isoreticulinine (**4**), but their low amounts precluded these assays.

### 2.4. Molecular Docking

All the new structures obtained from *H. reticulatum* had their theoretical acetyl- and butyrylcholinesterase inhibitory potential evaluated by molecular docking. The results of their binding interactions and orientation patterns with the active site gorge of AChE and BuChE are represented in Table 5.

**Figure 5:**
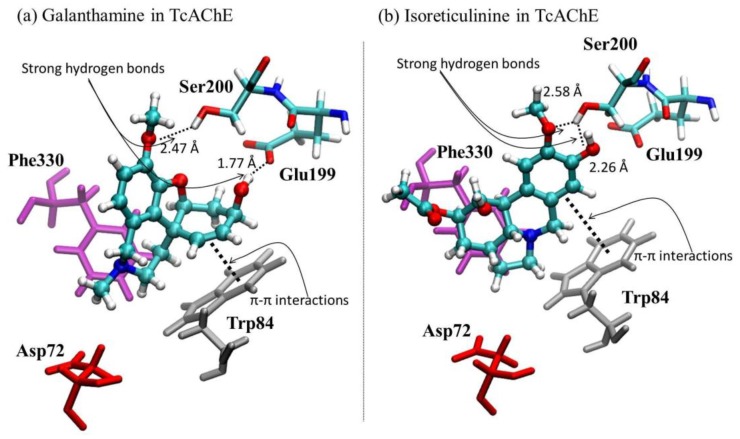
Graphical representations of (**a**) galanthamine-*Tc*AChE and (**b**) isoreticulinine*-Tc*AChE complexes.

The simulated molecular docking on the 1DX6 and 4EY7 structures showed that the alkaloid isoreticulinine (**4**) theoretically has a higher inhibitory effect against AChE than reticulinine (**3**), 6β-hydroxymaritidine (**1**) and 6α-hydroxymaritidine (**2**), but lower than galanthamine by 0.34 and 0.30 kcal mol^−1^, respectively. The docking results obtained for isoreticulinine suggest that the presence of a hydroxyl and an acetyl group at the C-1 and C-2 positions, respectively, theoretically improves the AChE inhibition on the 1DX6 and 4EY7 structures in comparison with reticulinine. Structural representations of the best conformation of the complexed active site of the *Tc*AChE with galanthamine and isoreticulinine (**4**) are depicted in Figure 5.

The molecular docking results reported in Table 5 are analyzed here in terms of different interactions between the alkaloids and active site of the *Tc*AChE protein: hydrogen bonds, π-π stacking and anionic interactions. Regarding hydrogen bonding, galanthamine showed the highest stability due to two strong interactions with the Glu199 and Ser200 residues, while the isoreticulinine complex presented only one interaction with the Ser200 residue. Both these alkaloids have a similar localization according to π-π stacking, interactions with Trp84, Phe330 and Asp72 residues, and anionic stabilization. The hydrogen bond interaction of reticulinine (**3**) is as depicted in Figure 5 for isoreticulinine (**4**), with a distance close to 1.9 A. The hydrogen bond interactions for 6β-hydroxymaritidine (**1**) and 6α-hydroxymaritidine (**2**) are similar, although anionic and π-π stacking interactions suggest a different, more distant localization, in accordance with the binding free energies reported in Table 5.

**Figure 6:**
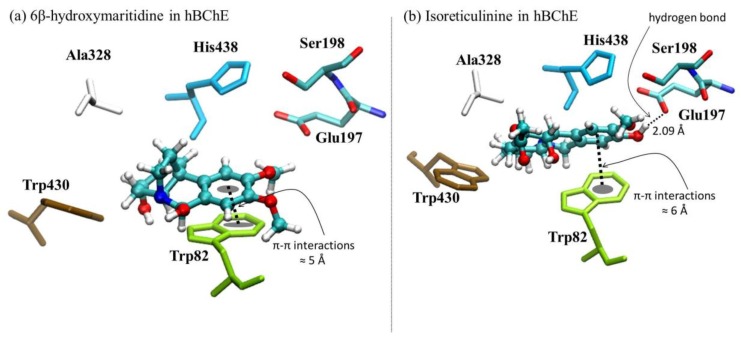
Graphical representations of (**a**) 6β-hydroxymaritidine-*h*BChE and (**b**) isoreticulinine *h*BChE complexes.

The molecular simulation of 6β-hydroxymaritidine (**1**) on the 4BDS structure theoretically showed more potent enzymatic inhibition against BuChE than 6α-hydroxymaritidine (**2**), isoreticulinine (**4**) and reticulinine (**3**), and interestingly, it was higher than galanthamine by 0.21 kcal mol^−1^. These results also indicate that 6α-hydroxymaritidine (**2**) theoretically has virtually the same inhibitory activity as galanthamine toward BuChE, differing by only 0.02 kcal mol^−1^. Furthermore, the docking results propose that the β-orientation of the hydroxyl group at the C-6 position in 6β-hydroxymaritidine (**1**) could improve the butyrylcholinesterase inhibition on the 4BDS structure by 0.23 kcal mol^−1^ compared to the α-orientation of the hydroxyl group at the C-6 position in 6α-hydroxymaritidine (**2**). Structural representations of the best conformation of the complexed active site of human BuChE with 6β-hydroxymaritidine (**1**) are presented in Figure 6.

In the case of BuChE, 4BDS in the PDB code, the crystallographic data show that tacrine, a molecule for anti-Alzheimer’s drugs targeting acetyl- and butyryl-cholinesterase, is stabilized by π-π stacking interactions between the aromatic rings and the Trp82 residue; additionally, this ligand is stabilized by the presence of hydrophobic interactions with Trp430, Ala328 and His438. On the other hand, similar to *Tc*AChE, the active site shows the presence of two available residues available for hydrogen bond interactions, Glu197 and Ser198. In terms of π-π stacking interactions, Figure 6 shows that 6β-hydroxymaritidine (**1**) is more stable than isoreticulinine (**4**). However, the localization of 6β-hydroxymaritidine in the active site is not appropriate for forming hydrogen bonds with the Glu197 and Ser198 residues. The results suggest that in an ideal BuChE inhibitor π-π stacking and hydrogen bond interactions should be in equilibrium, and among the tested molecules isoreticulinine was closest to this definition. In terms of hydrophobic interactions, all molecular docking analyses reported similar interactions between the alkaloids and Trp430, Ala328 and His438 residues.

The theoretical results obtained by molecular docking for 6β-hydroxymaritidine (**1**) and 6α-hydroxymaritidine (**2**) BuChE inhibition are not in agreement with the experimental assays. The difference between the BuChE structure used for the molecular docking (human) and experiments (equine serum), as well as the inability of these compounds to arrive to the active site gorge of BuChE may help understand the difference between the theoretical and practical results. Moreover, the use of an epimer mixture in the assays could also have contributed to the discrepancy between the theoretical and practical results.

According to the molecular interactions shown in the docking analysis, the isoreticulinine alkaloid (**4**) may be catalogued as a potential inhibitory molecule based on its very good interaction with the active site through strong hydrogen bonds in both of the proteins evaluated, AChE and BuChE. Additionally, the position of isoreticulinine alkaloid (**4**) on the gorge of the active sites showed that it can be stabilized by π-π stacking and hydrophobic interactions.

AChE is highly selective for ACh hydrolysis, while BuChE is able to metabolize different substrates [31]. AChE activity in the brain of Alzheimer's disease patients tends to decrease, while that of BuChE increases [32]. Consequently, the search for a structure able to simultaneously inhibit both enzymes may provide a better therapeutic response for Alzheimer’s disease than AChE-selective agents [33]. This information adds to the importance of the results obtained herein for isoreticulinine (**4**) by molecular docking.

## 3. Materials and Methods

### 3.1. General Experimental Procedures

About 2 mg of each alkaloid extract were dissolved in 1000 µL of MeOH and/or CHCl_3_ and injected directly into the GC-MS apparatus (Agilent Technologies 6890N coupled with MSD5975 inert XL, Santa Clara, CA, USA) operating in the EI mode at 70 eV. A Sapiens-X5 MS column (30 m × 0.25 mm i.d., film thickness 0.25 µm, Teknokroma, Barcelona, Spain) was used. The temperature gradient performed was the following: 2 min at 100 °C, 100–180 °C at 15 °C min^−1^, 180–300 °C at 5 °C min^−1^ and 10 min hold at 300 °C. The injector and detector temperatures were 250 °C and 280 °C, respectively, and the flow-rate of carrier gas (He) was 1 mL min^−1^. A split ratio of 1:10 was applied and the injection volume was 1 µL.

^1^H, ^13^C-NMR, COSY, NOESY, HSQC, and HMBC spectra were recorded on a Varian VNMRS 500 MHz (Palo Alto, CA, USA) and a Bruker 400 MHz Avance III (Billerica, MA, USA) equipped with CryoProbe Prodigy, using CDCl_3_ as the solvent and TMS as the internal standard. Chemical shifts are reported in units of *δ* (ppm) and coupling constants (*J*) expressed in Hz. CD, UV and IR spectra were recorded on Jasco-J-810 (Easton, MD, USA), Dinko UV2310 (Barcelona, Spain) and Thermo Scientific Nicolet iN10 MX spectrophotometers (Waltham, MA, USA), respectively. HR-ESI-MS spectra were obtained on an LC/MSD-TOF (2006) mass spectrometer (Agilent technologies, Santa Clara, CA, USA). Silica gel SDS chromagel 60 A CC (6–35 µm) (Carlo Erba Reagents, Val de Reuil, France) was used for VLC, and silica gel 60 F254 (Merck, Darmstadt, Germany) for analytics and preparative TLC. Spots on chromatograms were detected under UV light (254 nm) (Spectroline, Westbury, NY, USA) and by Dragendorff’s reagent stain.

### 3.2. Plant Material

Bulbs and leaves of *Hippeastrum reticulatum* Herb. were collected in Floresta Nacional de Goytacazes (Espírito Santo, Brazil) in May 2015. The samples were authenticated by Dr Julie H. A. Dutilh, Universidade de Campinas (UNICAMP, Campinas, Brazil). A specimen voucher (VIES 38724) has been deposited in the Herbarium VIES from Universidade Federal do Espírito Santo (UFES, Vitória, Brazil).

### 3.3. Extraction and Isolation

Fresh bulbs (1.5 kg) and leaves (400 g) of *H. reticulatum* were collected and macerated with MeOH (2 × 1.0 L) at room temperature for 4 days, the combined macerate filtered and the solvent evaporated to dryness under reduced pressure. The bulb and leaf crude extracts (117.3 and 53.5 g, respectively) were then acidified to pH 3 with diluted H_2_SO_4_ (2%, *v/v*). The neutral material was removed with Et_2_O (10 × 150 mL) and then extracted with EtOAc (3 × 150 mL) to provide the acid EtOAc extracts (0.60 and 0.44 g, respectively). The aqueous solutions were basified up to pH 9-10 with NH_4_OH (25%, *v/v*) and extracted with *n*-Hex (16 × 150 mL) to give the *n*-Hex extracts (0.14 and 0.02 g, respectively), which were followed by extraction with EtOAc (15 × 150 mL) to provide the EtOAc extracts (1.6 and 0.3 g, respectively) and finally extracted with EtOAc:MeOH (3:1, *v/v*) (4 × 150 mL) to provide the EtOAc:MeOH extracts (2.10 and 1.56 g, respectively).

The extracts were subjected to a combination of chromatographic techniques, including vacuum liquid chromatography (VLC) [34] and semi-preparative TLC. The general VLC procedure consisted of the use of a silica gel 60 A (6–35 µm) column with a height of 4 cm and a variable diameter according to the amount of sample (2.5 cm for 400–1000 mg; 1.5 cm for 150–400 mg). Alkaloids were eluted with *n*-Hex containing increasing EtOAc concentrations, followed by neat EtOAc, which was gradually enriched with MeOH (reaching a maximum concentration of 20%, *v/v*). Fractions of 10–15 mL were collected, monitored by TLC (UV 254 nm, Dragendorff’s reagent [35,36]), and combined according to their profiles. For semi-preparative TLC, silica gel 60F_254_ was used (20 cm × 20 cm × 0.25 mm) together with different solvent mixtures depending on each particular sample (EtOAc:MeOH, 9:1, *v/v* or EtOAc:MeOH, 8:2, *v/v*), always in an environment saturated with ammonia. Each known alkaloid was identified by GC-MS and the four new alkaloids were structurally elucidated by NMR.

The EtOAc leaf extract (0.3 g) was subjected to a VLC column (2.5 cm × 4.0 cm), starting the elution with 100% *n*-Hex, gradually increasing the polarity with EtOAc up to 100% EtOAc, and finally increasing the MeOH percentage in the mixture up to a ratio of EtOAc:MeOH (80:20, *v/v*), similar to the methodology used in *Hippeastrum aulicum* by Bessa and co-workers [37]. 580 fractions (10 mL each) were collected, analyzed by TLC and grouped in nine fractions. Fraction 5 (72.8 mg), eluted with EtOAc:MeOH (eluted with 95:05 until 90:10, *v/v*), was subjected to a semi-preparative TLC using 2 plates and a mobile phase consisting of EtOAc:MeOH (80:20, *v/v*) in an environment saturated with ammonia, and 15.60 mg of the epimer mixture **1** and **2** was isolated.

Exclusion chromatography was carried out using a Sephadex LH-20 column (2.5 cm × 40 cm) to clean and separate the alkaloids present in the EtOAc bulb extract (1.6 g). It was eluted with 100% MeOH, producing 147 fractions, each one containing about 2 mL, which were monitored by TLC and grouped in four fractions. Fraction 2 (801 mg) was chromatographed again in a Sephadex LH-20 in the same conditions, producing 120 sub-fractions, which were grouped in seven sub-fractions. Sub-fraction 2 (10.2 mg) was subjected to a TLC plate, applying 3 runs over a mobile phase consisting of EtOAc:MeOH (80:20, *v/v*) in an environment saturated with ammonia to obtain 1.09 mg of the isomers **3** and **4**.

### 3.4. Characterization of Compounds

*6β-Hydroxymaritidine* (**1**) and *6α-hydroxymaritidine* (**2**): Amorphous solid; [α]${}_{D}^{22}$ −11.22 (*c* 0.98, CHCl_3_); UV (MeOH) λ_max_ (log ε): 282.5 (3.64), 234.0 (3.92) nm; CD (MeOH, 20 °C) Δε*_250_* −340, Δε*_284_* +5938; IR *v*_max_ cm^−1^ 3341, 2958, 1609, 1512, 1464, 1406, 1268, 1246, 1222, 1192, 1141, 1113, 1043, 1017, 941, 866, 779, 746; ^1^H-NMR (CDCl_3_, 500 MHz) and ^13^C-NMR (CDCl_3_, 125 MHz) see Table 2; EIMS data shown in Table 1; HREIMS of [M + H]^+^ at *m/z* 304.1544 (calcd 304.1543).

*Reticulinine* (**3**) and *isoreticulinine* (**4**): Amorphous solid; ^1^H-NMR (CDCl_3_, 400 MHz) and ^13^C-NMR (CDCl_3_, 100 MHz) see Table 3; IR *v*_max_ cm^−1^ 2852, 1732, 1571, 1513, 1452, 1375, 1240, 1114, 1047; EIMS data shown in Table 1; [M + H]^+^ at *m/z* 334.1651 (calcd 334.1649).

### 3.5. Biological Activity

#### 3.5.1. Antiprotozoal Activity

In vitro tests for the biological activity of the epimers 6β-hydroxymaritidine (**1**) and 6α-hydroxymaritidine (**2**) against *Trypanosoma brucei rhodesiense* (trypomastigotes forms, STIB 900 strain), *Trypanosoma cruzi* (amastigotes forms, Tulahuen C4 strain), *Leishmania donovani* (amastigotes forms, MHOM-ET-67/L82 strain), and *Plasmodium falciparum* (intraerythrocytic forms, IEF, NF54 strain) and a cytotoxicity test against the mammalian L6 cell line from rat skeletal myoblasts were carried out at the Swiss Tropical and Public Health Institute (Swiss TPH, Basel, Switzerland) according to established protocols as described by Orhan and co-workers [38]. The reference drugs used in these assays were melarsoprol, benznidazole, miltefosine, chloroquine and podophyllotoxin, respectively.

#### 3.5.2. Acetylcholinesterase and Butyrylcholinesterase Inhibitory Activities

Cholinesterase inhibitory activities were analyzed as by Ellman and co-workers [39] with some modifications as by López and co-workers [40]. Fifty microliters of AChE or BuChE in phosphate buffer (8 mM K_2_HPO_4_, 2.3 mM NaH_2_PO_4_, 0.15 NaCl, pH 7.5) and 50 µL of the sample dissolved in the same buffer were added to the wells. The plates were incubated for 30 minutes at room temperature before 100 µL of the substrate solution (0.1 M Na_2_HPO_4_, 0.5 M DTNB, and 0.6 mM ATCI or 0.24 mM BTCI in Millipore water, pH 7.5) was added. The absorbance was read in a Labsystem microplate reader (Thermo Electron Corporation, Vantaa, Finland) at 405 nm after 10 minutes. Enzyme activity was calculated as a percentage compared to an assay using a buffer without any inhibitor. The cholinesterase inhibitory data were analyzed with the software Microsoft Office Excel 2010. The epimer concentrations used to calculate IC_50_ values were 10, 20, 40, 60, 80, 100 and 200 µg mL^−1^ in both AChE and BuChE assays.

### 3.6. Molecular Docking

The molecular docking simulations for 6β-hydroxymaritidine (**1**), 6α-hydroxymaritidine (**2**), reticulinine (**3**) and isoreticulinine (**4**) were performed to investigate the binding mode in the active site of three different enzymes, *Torpedo californica* AChE (*Tc*AChE), hBChE, and hAChE, proteins with PDB codes 1DX6 [41], 4BDS [42], and 4EY7 [43], respectively. The 3D structures of the alkaloids were drawn with the Chemcraft program [44] and then submitted to a geometrical optimization procedure using PBE0 [45] /6-311+g* [46] level of theory with the Gaussian 09 program [47]. All optimized alkaloids were confirmed as a minimum on the potential energy surface. The docking simulations for the set of optimized ligands were performed using the AutoDock v.4.2 program [48].

AutoDock combines a rapid energy evaluation through precalculated grids of affinity potentials with a variety of search algorithms to find suitable binding positions for a ligand on a given macromolecule. To compare the results from the docking simulations, the water molecules, cofactors, and ions were excluded from each X-ray crystallographic structure. Likewise, the polar hydrogen atoms of the enzymes were added and the non-polar hydrogen atoms were merged. Finally, the enzyme was treated as a rigid body. The grid maps of interaction energy for various atom types with each macromolecule were calculated by the auxiliary program AutoGrid choosing a grid box with dimensions of 60 × 60 × 60 Å around the active site, which was sufficiently large to include the most important residues of each enzyme. The docking searches for the best orientations of the ligands binding to the active site of each protein were performed using the Lamarckian Genetic Algorithm, (LGA) [49]. The LGA protocol applied a population size of 2000 individuals, while 2,500,000 energy evaluations were used for the 50 LGA runs. The best conformations were chosen from the lowest docked energy solutions in the cluster populated by the highest number of conformations. The best docking complex solutions (poses) were analyzed according to the potential intermolecular interactions (ligand/enzyme), such as hydrogen bonding and the cation-π, π-π stacking.

## 4. Conclusions

This is the first report about the alkaloid profile and biological activities of *H. reticulatum*. Twelve alkaloids were identified in this species, eight known and four new, 6α-hydroxymaritidine, 6β-hydroxymaritidine, reticulinine and isoreticulinine. The low cytotoxicity of the epimers 6β-hydroxymaritidine (**1**) and 6α-hydroxymaritidine (**2**) and the very good interactions of isoreticulinine (**4**) with the active sites of the enzymes AChE and BuChE by molecular docking suggests that *H. reticulatum* has high potential as a source of alkaloids with pharmacological activities. Finally, as the molecular docking results indicate that isoreticulinine alkaloid (**4**) is a promising molecule in the treatment of Alzheimer's disease, the synthesis of this compound and its analogues will be undertaken in future work in our laboratories.

## Figures and Tables

**Table 1:** Alkaloids identified in *H. reticulatum* by GC-MS.

alkaloid	RI	M^+^	MS
galanthamine (11)	2410.8	287 (84)	288 (20), 286 (100), 270 (15), 244 (27), 230 (15), 216 (37), 174 (32), 159 (11), 115 (16)
sanguinine (12)	2430.5	273 (100)	274 (16), 272 (77), 256 (23), 202 (31), 197 (15), 165 (14) 160 (45), 152 (15), 115 (19)
6β- and 6α-hydroxymaritidine (1) and (2)	2495.5	303 (20)	286 (9), 274 (12), 260 (21), 259 (100), 258 (12), 256 (20), 241 (30) 128 (16), 115 (20)
8-*O*-demethylmaritidine (6)	2522.4	273 (100)	274 (18), 230 (24), 203 (19), 202 (25), 201(89), 189 (55), 175 (23), 115 (19), 56 (20)
maritidine (5)	2528.5	287 (100)	288 (20), 244 (35), 217 (20) 216 (25), 215 (95), 203 (55), 189 (18), 128 (17), 115 (20)
11,12-dehydroanhydrolycorine (10)	2629.3	249 (59)	248 (100), 218 (1),190 (26), 163 (8), 137 (1), 123 (5), 95 (13)
*m/z* 264	2693.6	265 (76)	264 (100), 248 (18), 220 (12), 191 (14),178 (18)
11-hydroxyvittatine (7)	2732.5	287 (5)	259 (16), 258 (100), 242 (9), 214 (9), 212 (8), 211 (13), 186 (13), 181 (14), 128 (11)
lycorine (9)	2771.8	287 (18)	286 (10), 268 (19), 250 (16), 238 (7), 227 (78), 226 (100), 211 (6), 147 (5), 119 (3)
isoreticulinine (4)	2829.1	333 (<1)	291 (37), 290 (100), 274 (11), 272 (6), 256 (5), 228 (5), 147 (13)
reticulinine (3)	2133.4	333 (42)	332 (100), 316 (5) 290 (6), 272 (56), 256 (31), 244 (14), 216 (14), 147 (26)
*m/z* 294	2950.3	295 (87)	294 (100), 278 (10), 264 (3), 250 (7), 235 (3), 221 (5), 207 (3), 194 (5)
*m/z* 280	2978.6	281 (75)	280 (100), 264 (12), 250 (3), 236 (11), 219 (4), 207 (4), 194 (8), 178 (4), 167 (3)
2-methoxypratosine (8)	3071.1	309 (100)	310 (20), 294 (18), 284 (7), 266 (24), 251 (14), 236 (6), 164 (4), 152 (6)

**Table 2:** NMR data for compounds **1** and **2** (500 MHz for ^1^H and 125 Hz for ^13^C, CDCl_3_).

	1		2	
No.	*δ*_C_, type	*δ*_H_ (*J* in Hz)	*δ*_C_, type	*δ*_H_ (*J* in Hz)
1	130.7, *d*	6.59, *d* (10.0)	129.2, *d*	6.51, *d* (10.1)
2	127.9, *d*	5.98, *dd* (5.3 and 10.4)	128.4, *d*	6.02, *dd* (5.1 and 10.0)
3	63.2, *d*	4.32, *m*	62.1, *d*	4.37, *m*
4α	31.0, *t*	1.72, *dt* (4.0 and 13.7)	30.5, *t*	1.85, *dt* (4.0 and 13.4)
4β	31.0, *t*	2.14, *dd* (4.1 and 13.3)	30.5, *t*	2.29, *dd* (4.2 and 13.8)
4a	56.4, *d*	4.15, *dd* (4.1 and 13.6)	61.7, *d*	3.89, *m*
6a	125.2, *s*	*-*	123.4, *s*	-
6	88.8, *d*	5.35, *s*	86.6, *d*	6.12, *s*
7	111.8, *d*	6.89, *s*	110.7, *d*	7.03, *s*
8	148.0, *s*	*-*	148.4, *s*	-
9	148.9, *s*	*-*	149.2, *s*	-
10	105.3, *d*	6.83, *s*	105.1, *d*	6.78, *s*
10a	136.0, *s*	*-*	133.7, *s*	-
10b	44.2, *s*	*-*	44.8, *s*	-
11exo	39.9, *t*	2.03, *m*	39.8, *t*	2.03
11endo	39.9, *t*	2.03, *m*	39.8, *t*	2.03
12exo	47.4, *t*	3.37, *ddd* (3.8; 10.0 and 13.6)	41.2, *t*	3.19, *ddd* (3.5; 10.4 and 13.6)
12endo	47.4, *t*	2.87, *ddd* (6.5; 9.0 and 13.1)	41.2, *t*	3.94, *m*
OMe	56.1, *q*	3.90, *s*	56.1, *q*	3.90, *s*
OMe	55.9, *q*	3.87, *s*	55.9, *q*	3.87, *s*

**Table 3:** NMR data for compounds **3** and **4** (400 MHz for ^1^H and 100 Hz for ^13^C, CDCl_3_).

	3		4	
No.	*δ*_C_, type	*δ*_H_ (*J* in Hz)	*δ*_C_, type	*δ*_H_ (*J* in Hz)
1	70.6 *d*	6.01, *t* (3.9)	66.9 *d*	4.77, *t* (1.5)
2	68.4 *d*	4.15, *ddd* (2.8; 7.1 and 9.8)	72.1 *d*	5.12, *ddd* (2.5; 5.4 and 11.6)
3α	25.8 *t*	2.02, *m*	26.8 *t*	2.23, *ddd* (6.2; 11.8 and 13.4)
3β	25.8 *t*	2.02, *m*	26.8 *t*	1.95, *m*
4	36.7 *d*	2.68, *m*	36.4 *d*	2.68, *m*
4a	58.0 *d*	3.11, *dd* (5.9 and 10.4)	57.4 *d*	3.22, *dd* (5.9 and 10.7)
6α	51.4 *t*	4.28, *d* (16.7)	51.6 *t*	4.29, *d* (16.4)
6β	51.4 *t*	3.73, *d* (16.7)	51.6 *t*	3.74, *d* (16.9)
6a	127.7 *s*	-	127.7 *s*	-
7	112.7 *d*	6.67, *s*	112.9 *d*	6.68, *s*
8	144.0 *s*	-	144.0 *s*	-
9	145.4 *s*	-	145.4 *s*	-
10	106.4 *d*	6.73, *s*	106.4 *d*	6.82, *s*
10a	126.7 *s*	-	126.7 *s*	-
10b	33.1 *d*	2.68, *m*	34.3 *d*	2.59, *d* (10.5)
11α	29.7 *t*	1.85, *m*	29.9 *t*	2.04, *m*
11β	29.7 *t*	1.77, *m*	29.9 *t*	1.85, *m*
12α	52.7 *t*	3.39, *m*	52.9 *t*	3.39, *m*
12β	52.7 *t*	2.81, *m*	52.9 *t*	2.81, *m*
OMe	56.0 *q*	3.82, *s*	56.1 *q*	3.89, *s*
MeCOO	172.2 *q*	2.06, *s*	170.4 *q*	2.16, *s*
MeCOO	21.1 *q*	2.06, *s*	21.3 *q*	2.16, *s*

**Table 4:** In vitro antiprotozoal and cytotoxic activities of **1** and **2**. Values expressed in µg ml^-1^.

Parasite	*T. b. rhodesiense*	*T. cruzi*	*L. donovani*	*P. falciparum*	Cytotoxicity
Stage	Trypomastigotes	Amastigotes	Amastigotes	IEF (intraerythrocytic)	
Strain	STIB 900 (IC_50_ )	Tulahuen C4 (IC_50_ )	MHOM-ET-67/L82 (IC_50_ )	NF54 (IC_50_ )	L6 (IC_50_ )
melarsoprol	0.0010				
benznidazole		1.080			
miltefosine			0.091		
chloroquine				0.002	
podophyllotoxin					0.007
compounds **1** and **2**	30.68	66.11	>100	32.86	>100

**Table 5:** Estimated energies of reaction and inhibition constants for the new compounds in the active sites of AChE and BuChE.

Alkaloids	1DX6 ^a^ (kcal mol^−1^)	4EY7 ^b^ (kcal mol^−1^)	4BDS ^c^ (kcal mol^−1^)
6β-hydroxymaritidine (**1**)	−8.49	−9.44	−8.95
6α-hydroxymaritidine (**2**)	−8.25	−8.75	−8.72
reticulinine (**3**)	−8.88	−8.87	−8.10
isoreticulinine (**4**)	−9.02	−9.80	−8.12
galanthamine (**11**)	−9.36	−10.10	−8.74

^a^ 1DX6: *Torpedo californica* Acetylcholinesterase; ^b^ 4EY7: Human Acetylcholinesterase; ^c^ 4BDS: Human Butyrylcholinesterase.
